# Soldier, civilian, criminal: identifying pathways to offending of ex-armed forces personnel in prison

**DOI:** 10.1080/1068316X.2016.1181175

**Published:** 2016-05-11

**Authors:** Verity Wainwright, Sharon McDonnell, Charlotte Lennox, Jenny Shaw, Jane Senior

**Affiliations:** ^a^ Institute of Brain, Behaviour and Mental Health, University of Manchester, Manchester, UK

**Keywords:** Ex-armed forces, prison, offending, mental health, qualitative methods

## Abstract

Little is known about why some ex-armed forces personnel become involved in the criminal justice system, however, they represent the largest known occupational group in prison. In-depth interviews were employed to explore possible pathways to offending. Twenty ex-armed forces personnel in prison were recruited from five prisons in England. Data were analysed using a combination of thematic analysis and constant comparison methods rooted in grounded theory. Four predominant themes were identified: *experiences of trauma and adversity; belonging; impulsivity and creating a soldier*. Participants had experienced a number of traumatic incidents and adversity in their lives, encompassing pre, during and post-service but felt a sense of belonging in the armed forces. Participants demonstrated impulsivity in a number of areas with links to both their service in the armed forces and offending behaviour. The creation of the identity of ‘soldier’ was perceived to impact participants’ lives in a number of ways, including their offending, alcohol use and coping with trauma. The interplay of these themes and their potential impact on participants’ pathways to offending are discussed.

## Introduction

The majority of ex-armed forces personnel make the transition from military to civilian life successfully. However, an important minority become involved in the criminal justice system (CJS). Whilst this group is less likely to offend in comparison to the general population, they represent the largest known occupational group in prison (Howard League, 2011). It has been estimated that ex-armed forces personnel represent 3.5% of the prison population in England and Wales, most commonly serving sentences for violent and/or sexual offences (Defence Analytical Services Agency [DASA], now Statistics at MoD [Ministry of Defence], 2010). In response to a recent review of ex-armed forces personnel in the CJS (Phillips, 2014), more effort to identify this group, at all stages of the CJS pathway, has been recommended.

In the limited UK based literature available, explanations of why some ex-armed forces personnel end up in prison often focus on a potential link between military experience, such as combat exposure, and offending behaviour (NAPO, 2009). There is some evidence to support the link between deployment and offending. MacManus et al. (2013) found there was a higher rate of all offending post-deployment in their study looking at lifetime offending in 13,856 armed forces (serving and ex-service) personnel. Furthermore, MacManus et al. (2012) found that symptoms of post-traumatic stress disorder (PTSD), common mental health problems and heavy drinking in returning personnel were all associated with self-reported post-deployment risk for violent behaviour.

However, evidence for the link between military experience and offending remains limited and the pathways to offending for ex-armed forces personnel are likely to be more complex. For example, an inquiry by The Howard League (2011) concluded there were three, overlapping, categories of vulnerable personnel: those from socially disadvantaged backgrounds who had been involved in crime before joining the forces; those who experienced difficulties during service, such as mental health problems or physical injury; and those who had difficulty making the transition from military to civilian life. Three, inter-related, factors were reported to have a consistent presence in interviews with ex-armed forces personnel in prison: social exclusion and isolation, alcohol use and financial pressures. Reports such as this, supported by discrete health needs analyses to inform local service delivery (such as NHS [National Health Service] Cheshire Warrington and Wirral’s, 2013) make important contributions to our understanding of this population. However, there is a need for a robust, academically driven, evidence base of understanding.

Exploring why a proportion of ex-armed forces personnel are imprisoned is essential in order to inform both service provision for this group and strategies to reduce the likelihood of re-offending. This study aimed to explore the pathways to offending of ex-armed forces personnel in prison from their perspective.

## Method

This study was conducted as part of a larger mixed methods study that aimed to explore the experiences, and mental health needs of ex-armed forces personnel in prison. The quantitative arm of the study involved the administration of a range of standardised measures to assess general well-being, mental health problems (anxiety, depression and PTSD) and any drug and alcohol misuse. This was augmented by access to prisoner health and discipline records, to explore the level, type and severity of mental health and substance misuse needs in a sample of 105 former service personnel in prison.

### Participants

In-depth interviews were conducted with 20 ex-armed forces personnel in prison between June 2014 and April 2015. Purposive sampling was used to ensure individuals with a range of ages, type of service, rank and offence types were interviewed. As female ex-armed forces personnel in prison represent a considerably smaller population and are likely to have different pathways to offending, only male ex-armed forces personnel in prison took part in the study. Participants were recruited from five adult male prisons in England. Table 1 presents the demographic characteristics of the sample.

**Table 1.** Interview sample characteristics.

Characteristic	*N*	Characteristic	*N*
Age		Length of service	
Mean (SD) years	36 (11)	Mean (SD), years	7 (5)
Range	20–54	Range	1–19
Ethnic group		Offence type	
White British	18	Violence against the person	10
Mixed/multiple	1	Drugs offences	4
Black	1	Sexual offences	3
Marital status		Burglary	2
Single	10	Miscellaneous	1
Married	4	Recorded mental health diagnoses	
Long term relationship	3	None	9
Divorced	3	PTSD	4
Education level		Depression	4
O-Levels/G.C.S.E’s	12	Depression and anxiety	1
A-Levels/HND’s	1	Personality Disorder	1
Left school with no qualifications	7	Asperger’s	1
Branch		Number of deployments	
Army	15	None	7
Navy	4	1–2	8
RAF	1	3–4	4
Engagement		>4	1
Regular	18	** **	
Reservist	2	** **	

### Interviews

All interviews were conducted by the first author – VW. Participants were provided with an information sheet and had the study, and the interview process, verbally explained to them. Time was given to allow all participants the opportunity to ask questions about the study. Questions were responded to by VW and only once the participant was happy with the response was written informed consent obtained. Nineteen interviews were audio recorded with informed consent; one interviewee did not consent to the interview being recorded so hand written notes were taken instead. Interviews lasted for between 15 and 55 minutes, with an average length of 35 minutes. They were conducted in a private room in the prison, most commonly in the legal visit area, but also in rooms on the wings or in offices, depending on availability.

An interview guide was developed to explore the perceptions and experiences of participants across four areas: pre-service, during service (including any deployment experience), post-service and offending behaviour to explore pathways to offending. The interview guide is presented in the Appendix. However, the guide was used only for prompts when necessary. To encourage a more natural, conversational style of interview, no rigid format of questioning was adhered to. The same, open-ended, question began all interviews – asking participants to talk about their childhood and the time leading up to them joining the armed forces. All interviews then followed an individual, interviewee-led, trajectory with the interviews ending by asking respondents how they had found the interview process.

### Analysis

All interviews were transcribed verbatim by VW with the exception of the one none audio-recorded interview. The organisational support of NVivo 10 software (2012) was utilised. Data were analysed using a combination of thematic analysis and constant comparison methods (Green & Thorogood, 2004), a technique influenced by the grounded theory approach (Glaser & Strauss, 1967). Transcripts were read and re-read carefully by the researcher and notes, observations and codes applied to the data. From these initial codes a coding framework was developed to begin the process of grouping codes into categories. This process was both inductive and deductive by using both pre-defined categories (i.e. pre-service, during service, post-service and offending) and allowing new categories to emerge from the data.

Analysis of data began whilst interviews were on-going, informing the point data saturation was achieved and new interviews were not adding anything new to the analysis. This way, codes applied to transcripts could be continually compared and contrasted, new codes developed and existing categories merged. Coding reports were developed, recording all incidents of each code across the transcripts. The ‘one sheet of paper’ method was used. This method allows the researcher to find the ‘story’ or theme by summarising the data included within a code and including all perspectives on a single sheet of paper. This ensured that all coded extracts were included and compared in the analysis process and allowed broader themes to develop to provide an account of ‘what is going on in the data’ (Ziebland & McPherson, 2006).

All individual transcripts were also read by the second author (SM). The transcripts were then systematically discussed to allow emerging themes to be tested and agreed. Resulting themes were then discussed and agreed with all co-authors. This process also provided critical distance within the analysis process as the researcher was aware of the perspectives and preconceptions she may bring to the analysis. Issues raised in the themes were often linked, in that one issue may generate or exacerbate another. In an attempt to try and understand why a specific theme might occur, it was important to examine how and why it might be inter-linked and related to others; thus themes were analysed and interpreted separately before then considering their relationship with one another. The themes discussed are those reported most widely by participants and tell the ‘story’ of the data – that is, the pathways to offending of male ex-armed forces personnel in prison. All quotations are anonymised to prevent any possible identification of individuals.

## Results and interpretation

**Figure 1.**
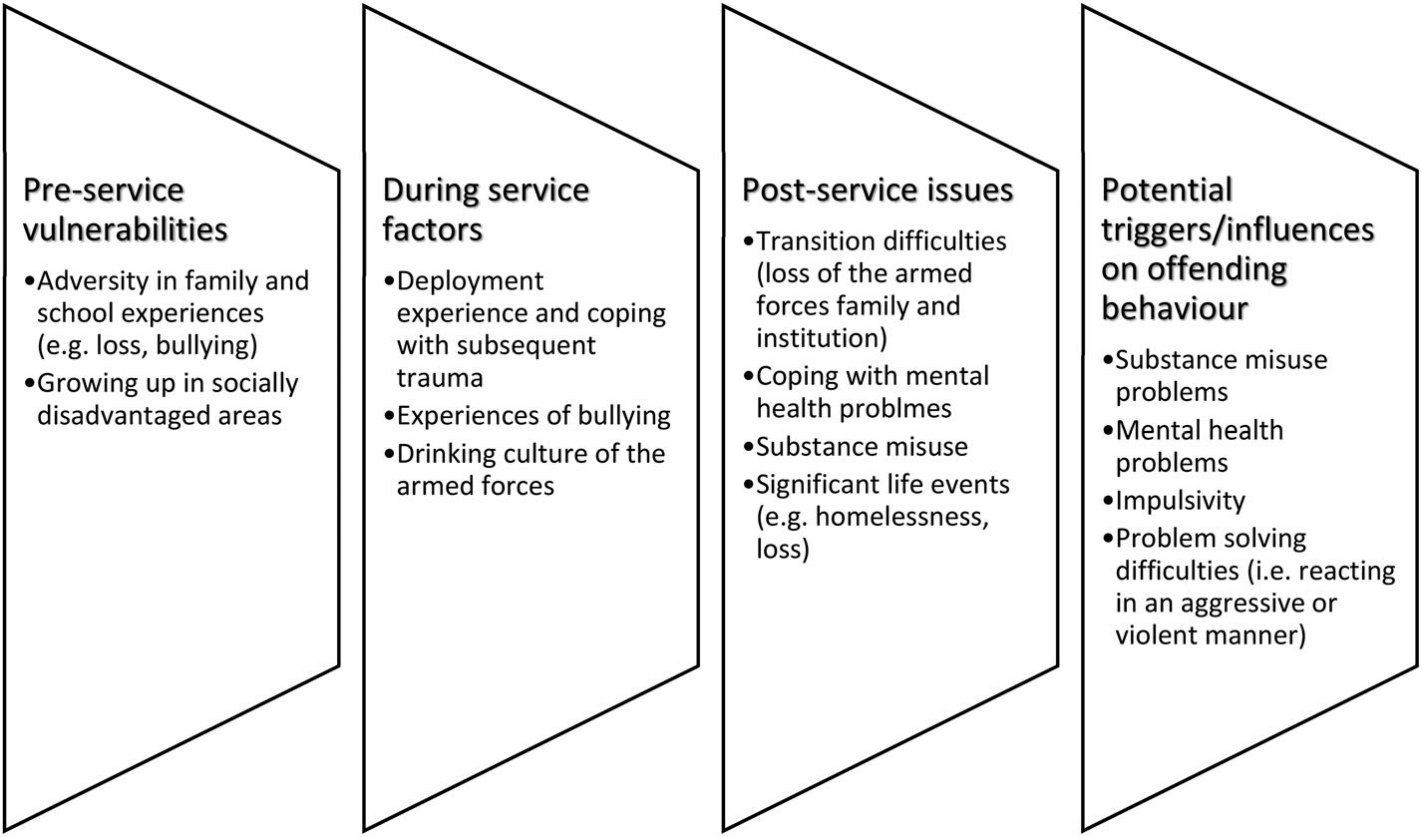
Factors influencing the offending pathway of ex-armed forces personnel.

The four major themes that related to ex-armed forces personnel’s perception of the pathway to their offending behaviour were: experiences of trauma and adversity; belonging; impulsivity and creating the identity of a soldier. The factors contributing to ex-armed forces personnel’s offending pathway, in this study, are shown in Figure 1.

### Experiences of trauma and adversity

Many of the men talked about experiencing difficult life events prior to joining the armed forces. Family life and school experiences were highlighted frequently, with many participants describing some level of disruption in their family circumstances including adoption, fostering, divorced or separated parents, and living with extended family relatives. In terms of school experiences, some noted experiencing bullying as well as being suspended or excluded from school. Some participants described experiencing a high level of trauma related to the loss (or multiple losses) of a parent(s) or guardian(s) at a young age, or experiences of physical or sexual abuse growing up. One man summarised his experiences prior to joining the armed forces: ‘ … I got expelled from school … I wasn’t really doing well at school due to my Dad dying so got expelled, that was in like year 10 … went back to sit my GCSE’s … I didn’t do very well on them … ’ (Participant 16).

The men spoke about a sense of hopelessness due to this early adversity and how it influenced decisions to join the armed forces. The men explained that they felt there were limited employment opportunities available to them, or they were not sure what alternatives existed, after leaving school. Some saw signing up as an escape to a different life, possibly away from any offending and antisocial behaviour they had already been involved in, explaining they were actively trying to avoid the ‘crime route’ or ending up in prison. As one participant explained:

I needed a new life to get away from it … where I’m from it’s bad you know what I mean, it’s really bad for knife crime, gun crime and you’re either with it or you’re against it so … I saw it as a way out … . (Participant 14)

For many, joining the armed forces appealed because of the perceived stability it would provide.

Despite the relief joining up was perceived as providing from past events, many of the men described further difficult life events occurring during their service in the armed forces. Nearly a quarter of participants relayed accounts of physical and psychological bullying experienced whilst serving, from both peers and superiors. As the following quote shows, for some the armed forces did not provide them with the stability they craved; instead it served to increase their sense of hopelessness:

Sometimes they’ll (other recruits) just single a person out and that kind of thing … more mentally than anything … in the first couple of weeks of training I got a bust nose … one night I remember … I went to sleep under my bed with my entrenching tool under my blanket. The entrenching tool is like a fold out spade, you know? So I slept with that under my bed … because that made me feel safe. (Participant 5)

Experiences of bullying occurred most often during initial training. Most commonly, however, discussions of trauma centred on deployment experiences.

Whilst most of the sample had experienced at least one deployment, some had been deployed on active service three or more times during their service. Countries deployed to included Northern Ireland, Bosnia, the Falklands, the Gulf and, more recently, Iraq and Afghanistan. Those who spoke about traumatic events were able to graphically recall details of those experiences. These usually concerned general combat situations such as facing enemy fire and those where participants had witnessed the death of a friend or colleague. The traumatic experiences were often spoken about in terms of how the men subsequently coped with the experiences reflecting that, at the time, they were not able to deal with them: ‘The effects of it are buried deep when you’re there, you push it down to carry on with what you’re doing … ’ (Participant 16) and ‘You need to … you can’t dwell on it, you couldn’t sit down and think about it too much at the time’ (Participant 19).

Traumatic and adverse experiences were not limited to before and during service. Some participants also discussed significant life events that occurred post-service. For two participants, this involved experiencing the loss of a loved one, and for one participant, a prolonged period of homelessness. Following discharge from the armed forces for mental health reasons following deployment, this man discussed his homelessness as partly a way of distancing himself from the Army and the difficulties re-integrating into society.

In addition to the impact such experiences could have on the general mental health and well-being of participants, exposure to traumatic experiences on deployment has been found to be associated with a higher risk of violent offending post-deployment (MacManus et al., 2013). These experiences may also serve to exacerbate the, already difficult, transition from military to civilian life. However, the potential impact of pre-service factors on the offending pathway cannot be ignored. A number of participants had suffered traumatic and adverse experiences prior to service and could be considered potentially vulnerable, and at risk of offending, before they joined the armed forces. Furthermore, some participants had already committed offences prior to their service. This is in line with the one of the vulnerable categories of ex-armed forces personnel identified by The Howard League inquiry (2011). Whilst, for some, service in the armed forces is recognised as likely to improve life chances and decrease the likelihood of interaction with the CJS (Fossey, 2010), for others it potentially only acts as an interlude in their offending trajectory. That experiences of trauma and adversity over time, from pre-service through to post-service, may impact on the pathways of offending for ex-armed forces personnel is demonstrated in Figure 1.

### Belonging

Men talked about ‘life’ in the armed forces as being a distinct culture to civilian life, becoming something much more than just a form of employment. Uniquely, participants often spoke about this culture as creating a strong sense of belonging – to the service, to peers and to the role they were trained to undertake. Relationships with peers were described in this context: ‘you’re one big family working together to achieve the same goal, you know everything is done in a team and that’s pretty good isn’t it?’ (Participant 17). However, this sense of belonging went beyond ties to immediate peers, it was also used to explain being a part of the wider institution that is the armed forces, and having found somewhere they felt they fitted in, as these two participants describe: ‘ … This is who I am, you know, this is my place in society’ (Participant 5) and ‘I felt like I was doing something, I felt like I was part of something’ (Participant 4).

Discussion of belonging most often came to light whilst participants considered their experiences of training. Participants often described how part of their training was forming a bond with peers and learning to work as part of a team, and how learning to trust and rely on those around them was purposively fostered. Many understood why this was promoted, predominantly in preparation for deployment: ‘they make you bond together to form a really strong team because they’re the people you’re going to be relying on all the time so if one of you is in trouble, you’re all in trouble…’ (Participant 7). Thus, whilst many participants described the process of training as both physically and psychologically difficult, they also understood its purpose in terms of their future role and the process was often discussed with some fondness.

The sense of belonging seemed to provide a feeling of hope for many of the men. Indeed, for some, life in the armed forces was the first time they felt they belonged and peers were often referred to as family. As Boyes (2010) stated, the development of a ‘Band of Brothers’ comradery is a necessity in an environment where personnel work, play, and rest together. As a consequence, leaving the armed forces is more than leaving a place of employment; for many it is a loss of family and a complete way of life. This loss, and the social isolation felt as a result, has been linked to an increased risk of common mental health problems (Hatch et al., 2013). For those who have experienced previous adversities, this may have served to intensify the sense of loss. Therefore, adjusting to civilian life not only requires them to find new employment and housing, but becomes a complete re-integration into society and dealing with the loss of the armed forces family. In terms of participants’ pathways to offending, whilst belonging was often viewed as a positive aspect of service, it potentially created a sense of dependency on the armed forces. Consequently, this makes the transition and adjustment to civilian life more difficult and thus has the potential to influence their offending behaviour.

### Impulsivity

Many participants described their propensity to impulsivity before, during and after service. For many, even joining the armed forces had been a wholly impulsive decision. The following two extracts demonstrate this:

… on my dinner hour one day…I just joined the Army. I went off to the careers office because I was bored and fed up, there’s nothing in (home town), I wasn’t going anywhere, nothing was happening, and yeah just literally went in my dinner hour. (Participant 4)

and ‘I ended up going with my mate one day…they said “what are you going to do when you leave school?” and I said “I don’t know” and the next minute I was signed up, that was it really’ (Participant 8). As well as impulsivity, these quotes also serve to further demonstrate the lack of direction experienced by a number of the men prior to joining the armed forces, adding further context to their seemingly recklessness around such a fundamental decision.

The leaving process for a number of participants also came across as impulsive. Half of the sample were early service leavers (ESLs), having served for under four years. The most common methods of leaving the armed forces were via disciplinary discharges and service no longer required (a form of administrative discharge). Impulsivity was often noted in the behaviour of participants leading up to discharge including going absent without leave (AWOL), committing offences whilst on leave, and using drugs:

I was partying on my leave and that and just had some Cocaine…when I went back there was a random spot drugs test and there was like 15 of us that got kicked out in the end, just a random drugs test. (Participant 8)

In some cases, even when participants made a conscious decision to leave the armed forces, they did so impulsively: ‘I got to the point I’d had enough…it was just a Tuesday and I just thought I’ve had enough of this and just drove home’ (Participant 7).

A number of the men reflected about the reasons why they left the forces. Mental health problems were the most commonly cited issues contributing to the circumstances, and impulsive behaviour, prior to discharge. Due to the rapid nature of discharge and the impulsive manner with which many of the men left the armed forces, there was often very little time to plan for post-service life. This is therefore likely to further compound the stress of both leaving the way of life they had become accustomed to, and where they felt they belonged, through an unsettling transition process into an unfamiliar way of life they had not properly considered.

Previous research has shown that higher impulsivity levels are linked to a number of adverse health and social outcomes including substance and alcohol misuse and psychiatric disorder (e.g. Moeller, Barratt, Dougherty, Schmitz, & Swann, 2001). Furthermore, a recent study by Mantzios (2014) found a positive, significant relationship between worry and impulsivity among military recruits. Worrying can be related to both anxiety and depression and therefore impulsive behaviour may be used as a way of coping with stressful or anxiety provoking situations. As a consequence, the impulsive behaviour of participants discussed both prior to, and during service in the armed forces, may be explained by this relationship. Indeed, it may also offer an explanation as to why impulsivity was also evident in the men’s offending behaviour. The most common offence type in the interview sample was violence against the person, followed by drugs and sexual offences. For most of the sample, this was their first time in prison and the men acknowledged that the nature of their offending often displayed a level of impulsivity. This was particularly the case for a number of the men with violent index offences: ‘it was a normal night and then I just…got into a fight and that was that…the end of my career…’ (Participant 15). In many cases alcohol use was discussed alongside offending behaviour, often to help explain their response to situations:

I was always told it’s better to talk your way out of a fight than to fight, and there’s a time and a place for that but if you’re full of alcohol you don’t think like that…that just goes out the window totally. (Participant 4)

However, the impulsive nature of participants’ offending could perhaps be linked to sensation seeking behaviour. The King’s Centre for Military Health Research report (KCMHR, 2010) found that deployment to Iraq was associated with an increase in risk taking behaviour post-deployment, such as driving too fast, not wearing a seatbelt, and driving under the influence of alcohol. The authors suggested that part of the reason for this was sensation seeking among returning personnel following the intensity of deployment. Sensation seeking may also be linked to substance misuse. Alcohol is recognised as being deeply rooted within the culture of the armed forces (Forbes, Fear, Iversen, & Dandeker, 2011) and previous research has shown a potential link between substance misuse and offending behaviour; Tsai, Rosenheck, Kasprow, and McGuire (2013) found that, of 17,204 US ex-armed forces personnel in prison, nearly half reported using substances (drugs, alcohol or drugs and alcohol) at the time of their offence.

### Creating a soldier

The process of creating and becoming a soldier, and the impact this had on other areas of their lives was a commonly discussed theme. Three subthemes were identified: programming, coping strategies and institutionalisation.

#### Programming

When reflecting on the reasons for their offending, some participants felt that their response to certain situations, and a level of violence and aggression, had been cultivated during their service. Training was described by one participant as: ‘…it was all breaking you back down to basics and then working you up as a soldier’ (Participant 7). Indeed, a number of participants referred to having been ‘programmed’ or ‘moulded’ to act and respond to situations in a particular manner during training as part of the process of becoming, and the armed forces creating, a soldier.

Some reflected on a possible link between their offending and their armed forces training: ‘I don’t know if it has anything to do with the Army…but it just…they are kind of training you to be violent, to be aggressive, to not really think about the consequences of it…’ (Participant 5). Some participants reflected how this training, whilst useful in the context of deployment, could have a negative impact in civilian life: ‘if it comes to an ex-soldier…there’s only one way he’s going to go when his back is against the wall…and that’s to come out fighting…’ (Participant 4) and

What’s that saying? We should be put in a glass cabinet and only broken out in the event of war because you miss the adrenaline, so I’m going to get pissed and I’m going to get into fights you know…. (Participant 14)

In effect, the men felt the training they had received, which was also used to build camaraderie and trust between recruits, also programmed them to respond to situations in a certain way. This involved breaking down how a person would ‘normally’ respond to situations as a civilian, and re-programming them as soldiers.

The perception by participants of the potential link between their training and offending are in line with what are commonly thought to explain offending, particularly violent crimes, by ex-armed forces personnel. That is, that they have had combat exposure and are trained in the use of violence.

#### Coping strategies

The use of alcohol in the armed forces has been seen as both a bonding tool to develop unit cohesion and as a coping device (Jones & Fear, 2011). This was echoed in this study with participants discussing a drinking culture in the armed forces as part of the development of their identity of a soldier: ‘well it’s (what) you do- you work hard, you play hard, and normally the play hard involves drinking…’ (Participant 4) and ‘if you’re not working you’re on the piss because it’s big boys rules’ (Participant 14). For those with mental health diagnoses, alcohol was also often used as a coping strategy, with participants often citing that increased alcohol intake made them realise they were suffering with mental health issues, particularly for those with PTSD on return from deployment. On reflection they related their increased alcohol use as a way of coping and blocking the symptoms that they were experiencing: ‘I just started drinking a lot- a lot more than usual…and it just went downhill from there…I was not drinking for social reasons anymore but to…to cover up my problems…’ (Participant 16). Levels of alcohol misuse have been found to be substantially higher amongst armed forces personnel in comparison to the general population (Fear et al., 2010), and often at a level considered harmful for health (Fear et al., 2007). These discussions by participants not only highlighted the non-functional, but normal, coping strategies used by some ex-armed forces personnel, but also the potential influence of mental health problems and alcohol misuse in relation to offending.

Participants also perceived their soldier identity as affecting how they dealt with subsequent trauma and mental health problems. Many explained they felt unable to seek support to cope with trauma or the mental health problems they were experiencing. This was most often discussed in the context of returning from deployment. Concern about being perceived as being ‘weak’ was often used to explain the reasoning behind not seeking help: ‘…you don’t want to be seen to need help. You want to be seen as a strong person who can just get on with life and take everything they throw at you’ (Participant 1). Another participant coherently linked in the concept of having being programmed, and their soldier identity, as having an effect on his help-seeking behaviour: ‘(it’s) the way I’m programmed, the way the Army want you to be. You’re trained to be proud and see yourself as strong…’ (Participant 14). This is in line with research by Iversen et al. (2005) who found that the most common reason for ex-armed forces personnel not seeking help was a sense of resilience, a belief that they should be able to deal with the problems they were experiencing themselves.

#### Institutionalisation

Attached to the identity of ‘soldier’ was a level of institutionalisation. One of the most common difficulties experienced in the transition from soldier to civilian was adjusting to the lack of structure and routine in civilian life:

It just wasn’t the same out. Your life falls into place in the Army. Everything’s done and it has to be done. But when you’re out, nothing gets done. You have to do it all yourself. They’ve done if for you your whole life so…how can I? (Participant 3)

and ‘Obviously when you’re in the Army there is a strict routine and then you come out and you’re not used to all the freedom and being on civvy street was so different’ (Participant 8).

Furthermore, following the loss of the soldier identity, and no longer feeling they belonged to the armed forces, many relayed a sense of feeling ‘used’. This was often explained in relation to deployment. Whereas prior to deployment it was often perceived as doing their job and fulfilling their purpose, after leaving the armed forces a number of participants cited feeling under-valued or ‘just a number’. Whilst some participants continued to feel pride at their deployment experience, a number felt their perception of deployment, and war, had changed. Feeling betrayed, and to an extent, abandoned, by the armed forces also came across when participants described how they felt unsupported during the leaving process and the transition to civilian life: ‘No like it was once you’re out you’re a civvy and when it came to it it was just you’re out now…that’s it…spat out by the system aren’t you?’ (Participant 9) and ‘That’s it, there’s no assistance or nothing – it’s just we’ve (the armed forces) wiped our hands of you…’ (Participant 19). Indeed, adjusting to civilian life was one of the biggest difficulties reported by participants, often due to the lack of structure and routine. This shows a level of institutionalisation within the armed forces. This is perhaps more often the case for those who felt a sense of belonging, and likely to increase feelings of abandonment. In addition to the loss of the armed forces ‘family’ and feeling of belonging, the loss of participants’ soldier identity could perhaps increase the difficulty of transitioning to civilian life, and coping with the number of life changes associated with that transition.

How ‘creating a soldier’ impacts on the offending pathway of ex-armed forces personnel in this study is multifaceted. In addition to the potentially enhanced transition difficulties as a consequence of institutionalisation, participants felt they had to an extent been programmed to respond to situations in a certain way as a consequence of their training. The Crime Survey for England and Wales (ONS [Office of National Statistics], 2015) revealed 53% of violent incidents were alcohol related. Heavy drinking and the use of alcohol to cope with problems, compounded further by the barriers to seeking help that participants’ perceived, are also likely to have an influence on the offending pathway of this group.

## Conclusions

Experiences of trauma and adversity, belonging, impulsivity and creating a soldier were identified as major themes within the data that ‘told the story’ of the pathways to offending from the perspective of these participants. Previous research has largely focused on the potential link between deployment experience and offending and the present study would suggest that from the perspective of ex-armed forces personnel in prison, their military experiences, particularly their training for and experience of deployment, were perceived to have some bearing on their offending behaviour. This was mostly discussed in terms of how they had been taught to respond to situations. However, the pathways to offending did come across as often being more complicated, incorporating a number of factors, both pre and post, as well as during service, and this was recognised by most participants. It is recognised that most ex-armed forces personnel make the transition from military to civilian life successfully and that service in the armed forces can offer stability, skills training and, in many cases a long and enjoyable career. These things may be protective factors for many personnel.

However, a thread of hopelessness ran through all of the themes presented here. The men discussed traumatic experiences and adversity experienced prior to their service, with many feeling that this gave them two choices: the armed forces or crime. This suggests that, in some respects, this group is not different from the general prison population in that prisoners predominantly come from socially disadvantaged backgrounds (Social Exclusion Unit, 2002). For many of the men the armed services created a sense of hope through belonging to this family. Nonetheless the dependency created by this and some of the associated dysfunctional behaviours (including alcohol, the need to maintain a macho image and exposure to violence contributing to the development of their identity as a soldier) have implications for their adjustment to civilian life and offending. Therefore, whilst not entirely dissimilar from the general prison population, ex-armed forces have specific idiosyncrasies as a consequence of their service experience that are likely to impact on their offending behaviour.

That the stigma of mental health issues is seemingly compounded by the masculine culture of the armed forces is not a new finding. Previous research has reported that stigma attached to mental health difficulties is a concern for serving personnel (Iversen et al., 2005). The armed forces have used a number of approaches to try to tackle this, including the Trauma Risk Management programme (TRiM) which is now used by many units (Greenberg, Langston, Iversen, & Wessely, 2011). This programme seeks to challenge the stigma attached to mental health issues (such as it being a sign of weakness), provide support and education, and identify and refer at risk personnel. These are important changes but to date there is little evidence of the effectiveness of these programmes (Murphy & Busuttil, 2014).

This study provides an insight into some of the potential difficulties providing support to this group may entail. Prison may provide an opportunity for former personnel to more easily access the help that some evidently need. To do this, we need to understand more specifically about the help-seeking behaviour of this group in prison. For instance, research has found that some former service personnel in the community would prefer to receive help from those with military service knowledge and/or expertise (Iversen & Greenberg, 2009).

The results of this study suggest that, when leaving the armed forces, some may need additional support, over and above that currently available, to make a successful transition to civilian life. Currently, when personnel make the decision to leave the armed forces they are referred to Career Transition Partnership (CTP), a partnership between a private sector career development company and the Ministry of Defence to prepare personnel for resettlement. This process is started well in advance of leaving service. To be eligible for the full package of service provision, personnel need to have served for six years or more. As mentioned, half of the current sample were ESLs and would therefore likely have received a much less comprehensive level of support based on length of service. In his review of the transition process for service personnel, Lord Ashcroft (2014) made a number of recommendations including a call to improve this resettlement package. Other recommendations promoted included a focus on a post-armed forces career from early on through the development of a personal development plan. Additionally, the need for more independence regarding day to day management of money and budgeting to be promoted during service would better equip personnel post-service. An update on the review has recently been published (Lord Ashcroft, 2015). This suggested that whilst progress had been made in a number of areas, including some change to the CTP provision, there was still much to do. This study would concur that the benefits of a smoother transition for a number of personnel cannot be underestimated. Improving the transition process would not only maximise the chances of adjustment to civilian life, but also may help minimise the potential ‘programming’ effect of service.

This study highlights a number of practical concerns. The impulsivity with which a number of participants joined the armed forces, and the level of vulnerability they import from pre-service experiences, need to be considered by those recruiting into the armed forces. However, there is no easy answer and the debate surrounding enhanced recruitment screening is nothing new (Wessely, 2005). It should be borne in mind too that a high percentage of non-veteran prisoners also show impulsivity. Cognitive-behavioural domains, such as problem solving and impulsivity, are the focus of offending behaviour programmes in prison aimed at reducing re-offending rates in England and Wales (Ministry of Justice, 2013). Programmes like Enhanced Thinking Skills (ETS) and, more recently, the Thinking Skills Programme (TSP), have shown effectiveness at reducing reoffending (Gobbett & Sellen, 2014; Ministry of Justice, 2010). It is unlikely these particular programmes would need to be specifically tailored to meet the needs of ex-armed forces personnel so there is scope for the impulsive behaviour evidenced in this study to be addressed for these personnel whilst in prison.

The links made between alcohol and offending, as well as the use of alcohol to cope with mental health problems, emphasise that strategies to combat the high level of drinking amongst armed forces personnel also need continued development. Indeed, criticisms of the armed forces education strategies to combat alcohol misuse and calls for improvement have recently been made (Kotecha, 2015; Owen & Crook, 2014). Services in the community providing support to veterans also need to understand the interplay of such factors for ex-armed forces personnel and their offending behaviour. As with impulsivity, there is a high prevalence of alcohol misuse in the general prison population (Fazel & Danesh, 2002). However, the limited service provision for this group whilst in custody, and lack of funding, have been criticised (HM Inspectorate, 2010). The Inspectorate report found that alcohol needs were less likely to be assessed or met in prison in comparison to those with drug misuse problems. Alcohol misuse is a public health priority and improving service provision in the community and prison continues to be reviewed (Public Health England, 2014).

This study adds weight to the findings of the inquiry by The Howard League (2011) in recognising the complexity of inter-twined factors that contribute to the pathways of offending of ex-armed forces personnel. A strength of this study is that the interview sample is both varied and representative of the larger sample of ex-armed forces personnel in prison recruited to the overall mixed methods study. Furthermore, military service of all participants was verified prior to interview. However, those interviewed had all willingly identified, when asked by prison staff, as being ex-armed forces. The results are therefore not necessarily representative of those who, for a number of reasons, have not identified themselves as having previously served in the armed forces. For instance, previous research has noted difficulties with the more commonly used term ‘veteran’ (Burdett et al., 2013) that many prison establishments use interchangeably with ‘ex-armed forces personnel’.

This qualitative study aimed to explore the pathways to offending of ex-armed forces personnel in prison, from their perspective, and identified a complex interplay of factors. Ex-armed forces personnel are less likely to offend than the general population (Statistics at MoD, 2010) but, as Murray (2013) suggests, they are a group who have a specific set of experiences and circumstances that need to be understood to reduce their risk of offending. This enables us to begin to build a knowledge base with which we can ensure appropriate support can be provided to this group and reduce the likelihood of them reoffending.

## Appendix: Interview guide

1. Introduction and greeting (aim to make participant at ease)
  - Re-introduce self and study
  - Explain the format of the interview and the difference between their participation in Part 1 and this interview (are able to talk freely, audio recorded to ensure accuracy)
  - Explain they do not need to answer anything they do not want to and can stop the interview at any time
2. First, I’d like to know a bit more about you and your background before you joined the forces. You previously told me you grew up in____, can you tell me a bit about your childhood/when you were growing up?
  - Did you experience any difficulties whilst growing up?
  - Did you live with your parents?
    o Explore relationship with family
  - How did you find your time at school?
3. Leading on to your time in the forces, you told me you joined when you were ____ years old, can you tell me a bit about why you decided to join the armed forces?
  - When and why did you decide you wanted to join the forces?
  - Before you joined, what did you think joining the armed forces would bring to your life?
  - Did your experiences in the armed forces live up to the expectations you had before you joined?
4. I’m interested in what your experiences of comradeship in the forces were…
  - Can you describe your relationship with your superiors?
  - Can you describe your relationship with your peers?
  - Did you ever experience any difficulties during your time in the forces?
5. **For those who identified in part 1 as having experienced AT LEAST ONE major deployment:** I’d now like to talk about your experiences around deployment. You previously told me you had experienced____ deployments while you were in the armed forces…
  - Did you feel you had enough training and were ready for deployment?
  - Did you experience any traumatic incidents?
    o What support did you receive following this?
      ▪ Explore accessibility of support and barriers to support
  o How did this impact on the remainder of the deployment?
  • How did you find the support from the armed forces whilst you were deployed?
    o Did you worry about what was happening at home whilst you were deployed? *(Explore support to family)*
    o Explore support received from family/partner whilst deployed
    o Explore support from superiors/leadership whilst deployed
  • How did you find homecoming after deployment?
    o Did you experience any mental health problems? If so, what did you do about these? Did you receive any support?
      ▪ Explore accessibility of support and any barriers
  o Explore issues around feelings of guilt
  o Explore issues around sensation seeking *(driving too fast, alcohol/drug consumption)*
  o Explore issues around any aggressive behaviour
  o Explore relationship issues
  - Did your experiences of deployment impact your decision to leave the forces?
6. You previously told me you left the armed forces because____, can you tell me a bit more about the circumstances around why you left and how you felt at the time?
  - How did you find the transition from military life to ‘civvy’ street?
    o Explore relationship issues
    o Explore financial issues
    o Explore issues around housing
    o Explore issues around employment
7. Overall, what impact do you think serving in the armed forces had on your life?
  - Any negative aspects?
  - Any positive impact?
  - What impact do you think being in the forces had on your offending behaviour?
  - What impact did service have on your mental health?
  - What impact did service have on your family? (Partner, children)
8. Can you tell me a bit more about the time leading up to the offence you are in prison for now?
  - What factors do you think influenced your behaviour at that time?
  - How did you feel at that time?
9. What impact has being in prison had on how you feel about yourself?
  - Explore effect on sense of pride/self-esteem
  - Did you worry about identifying that you had served in the forces when you came into prison, and if so, why?
    o To prison staff?
    o Other prisoners?
10. That’s the end of my questions, is there anything else you would like to discuss/tell me about? How have you found the interview?
11. Thank participant and debrief
